# Genome-Wide Analysis Reveals Coating of the Mitochondrial Genome by TFAM

**DOI:** 10.1371/journal.pone.0074513

**Published:** 2013-08-26

**Authors:** Yun E. Wang, Georgi K. Marinov, Barbara J. Wold, David C. Chan

**Affiliations:** 1 Division of Biology, California Institute of Technology, Pasadena, California, United States of America; 2 Howard Hughes Medical Institute, California Institute of Technology, Pasadena, California, United States of America; University of Texas Health Science Center at San Antonio, United States of America

## Abstract

Mitochondria contain a 16.6 kb circular genome encoding 13 proteins as well as mitochondrial tRNAs and rRNAs. Copies of the genome are organized into nucleoids containing both DNA and proteins, including the machinery required for mtDNA replication and transcription. The transcription factor TFAM is critical for initiation of transcription and replication of the genome, and is also thought to perform a packaging function. Although specific binding sites required for initiation of transcription have been identified in the D-loop, little is known about the characteristics of TFAM binding in its nonspecific packaging state. In addition, it is unclear whether TFAM also plays a role in the regulation of nuclear gene expression. Here we investigate these questions by using ChIP-seq to directly localize TFAM binding to DNA in human cells. Our results demonstrate that TFAM uniformly coats the whole mitochondrial genome, with no evidence of robust TFAM binding to the nuclear genome. Our study represents the first high-resolution assessment of TFAM binding on a genome-wide scale in human cells.

## Introduction

Mitochondria are essential eukaryotic organelles, serving as the epicenter of ATP production in the cell through oxidative phosphorylation. To perform this bioenergetic function, mitochondria utilize gene products encoded by the mitochondrial genome, a circular DNA that is 16.6 kb long. This genome is organized into DNA/protein structures termed nucleoids [1]. Mitochondrial DNA (mtDNA) encodes thirteen components of the electron transport chain, as well as 22 tRNAs and two ribosomal RNA genes. These gene products are essential for the proper function of the respiratory chain, and therefore maintenance of mtDNA levels and sequence fidelity is essential for cellular bioenergetics. In a human cell, there are hundreds to thousands of copies of the mtDNA genome [2,3]. Damage or depletion of mtDNA causes numerous inherited disorders, including Alpers’ Disease, ataxia neuropathy spectrum, and progressive external ophthalmoplegia [4,5]. Furthermore, loss and damage to mtDNA has been implicated in cardiovascular disease [6–9], diabetes [10–12], neurodegenerative disorders such as Alzheimer’s [13,14], and aging [15,16]. Strikingly, increasing mtDNA copy number promotes cell survival or function in many models of disease associated with decreased mtDNA abundance, such as diabetes [12,17], aging [18], Alzheimer’s [19], and Parkinson’s [20,21]. Thus, it is critical to understand how mtDNA copy number and integrity are maintained.

Mitochondrial transcription factor A (TFAM) is a DNA binding protein that plays multiple roles in regulating mtDNA function. As a sequence-specific transcription factor, it binds upstream of the light strand promoter (LSP) and heavy strand promoter 1 (HSP1) to activate initiation of transcription. At these sites, the footprint of TFAM binding is ~22 bp long [22,23]. As a result, TFAM is essential for production of gene products from the mitochondrial genome. In addition, TFAM is required for normal mtDNA copy number, because RNA primers generated from LSP are used to prime mtDNA replication [24,25]. Mice heterozygous for a knockout of TFAM exhibit not only an expected reduction (22%) in mitochondrial transcript levels in the heart and kidney, but also a universal 34% reduction in mtDNA copy number across all assayed tissues. Furthermore, homozygous knockout mice have no detectable levels of mtDNA and die during embryogenesis [26], highlighting the importance of TFAM in maintenance of mtDNA levels and in cellular and organismal viability.

Apart from its sequence-specific functions, TFAM is thought to organize the mtDNA genome by coating it in a nonspecific manner. Although how TFAM packages mtDNA is not well-understood, it is known to bind nonspecifically to DNA [27] and is estimated to be sufficiently abundant to coat the genome completely [28–30]. One model suggests that nonspecific binding radiates from the TFAM LSP binding site, which acts as a nucleation site for subsequent cooperative binding in a phased pattern to yield an inter-genome homogeneous pattern of binding [31,32]. The packaging function of TFAM appears to have important consequences for maintenance of the mtDNA genome. A TFAM variant that is deficient in transcriptional activation but competent in DNA binding is capable of preventing mtDNA depletion [33]. Therefore, as a prominent component of mtDNA nucleoids, TFAM appears to coat the mitochondrial genome, perhaps protecting it from turnover or deleterious damage.

Despite the importance of the associations of TFAM with mtDNA in the maintenance of mtDNA integrity and in cellular viability, these interactions have only been visualized in vivo at low resolution [34]. Therefore, to capture a high-resolution profile of TFAM-mtDNA interactions across the entire mitochondrial genome, we performed chromatin immunoprecipitation followed by massively parallel sequencing (ChIP-seq) for TFAM in human HeLa cells.

## Results

### Detection of TFAM-DNA interactions using ChIP-seq

**Figure 1:**
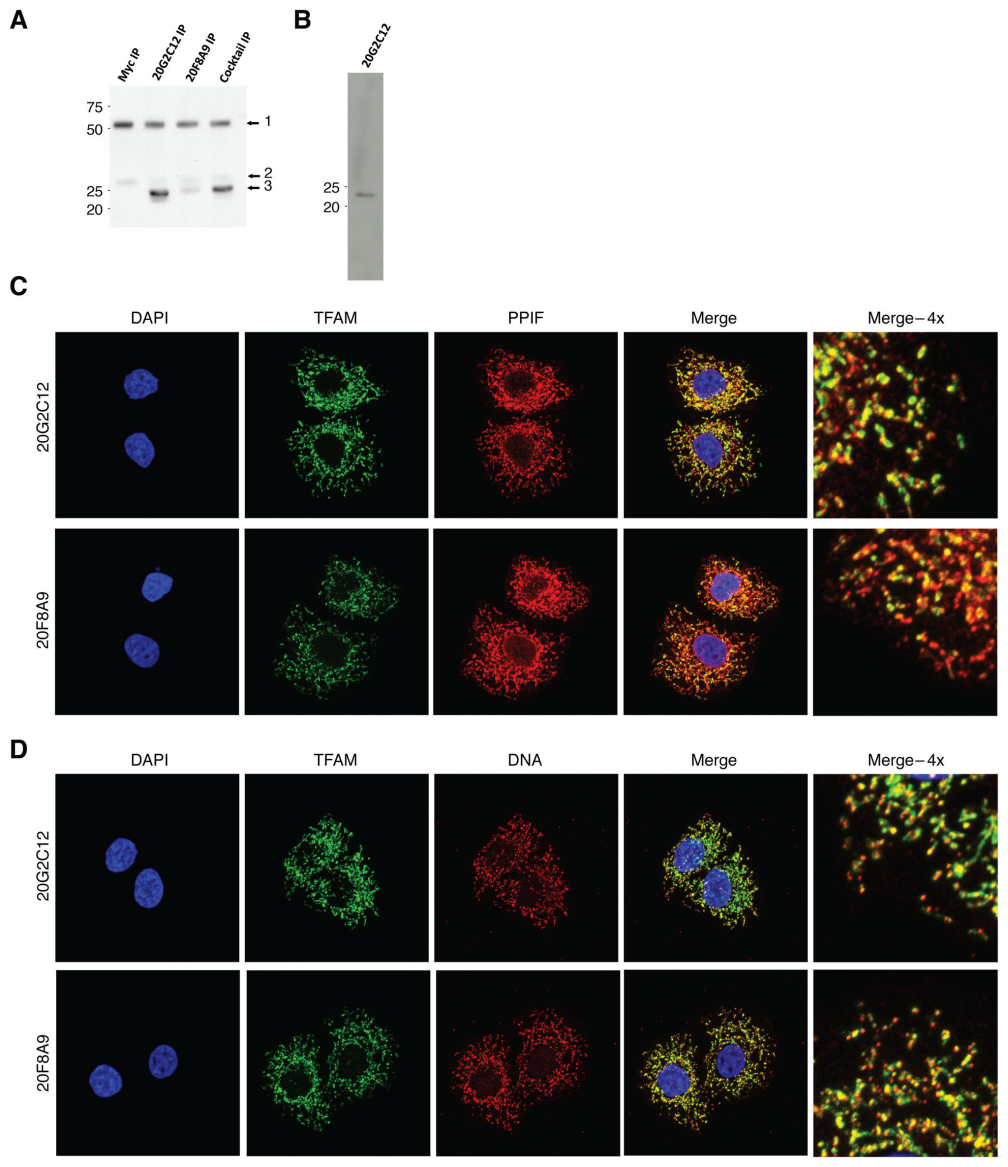
Characterization of TFAM monoclonal antibodies. (A) Immunoprecipitation of TFAM from cell lysates. HeLa cell lysate was applied to sheep anti-mouse Dynabeads conjugated to anti-Myc, 20G2C12 TFAM antibody, 20F8A9 TFAM antibody, or a 50/50 mixture of 20G2C12 and 20F8A9 TFAM antibodies. The labeled bands are: 1) Antibody heavy chain; 2) antibody light chain; 3) TFAM. (B) Western blot using the 20G2C12 antibody detects a ~23kDa band. (C and D) Immunocytochemistry showing TFAM localization. Mitochondria were identified by PPIF staining; mtDNA was identified by anti-DNA staining. There was no evidence for nuclear localization of TFAM using either antibody.

To characterize TFAM binding to both the mitochondrial and nuclear genomes in an unbiased manner, we performed ChIP-seq targeting TFAM in HeLa cells. Because ChIP-seq data is highly dependent on the use of high-quality antibodies, we generated two new TFAM monoclonal antibodies (20G2C12 and 20F8A9) that efficiently immunoprecipitated TFAM (Figure 1A). Both of these antibodies gave clean mitochondrial and nucleoid signals in immunofluorescence experiments with cultured HeLa cells (Figure 1C,D). The 20G2C12 antibody also performed well in Western blots of whole-cell lysates, recognizing a single protein band of ~23 kDa (Figure 1B).

Given the high efficiency of 20G2C12 in immunoprecipitating TFAM, as well as its high specificity, we used it to capture TFAM-associated DNA fragments for ChIP-seq analysis. DNA was sonicated prior to immunoenrichment and size-selected prior to library building so that the average fragment length of the final library was centered around 200 bp, a fragment distribution allowing for high-resolution deconvolution of binding events. We generated 3 replicates and matching controls. The sequencing depth of all samples was between 18 million and 48 million mappable reads, which is generally sufficient for comprehensive identification of transcription factor binding sites [35].

A common concern with ChIP-seq datasets is the variability of enrichment for true binding events as compared to background. In a typical ChIP-seq experiment, a minority of sequencing reads originates from binding events, with the majority representing random genomic DNA. Even for the same DNA binding factor, large variations in the strength of enrichment can be observed, and therefore it is critical to assess the degree of enrichment before downstream analysis. A number of ChIP-seq quality control metrics have been developed [35] for nuclear transcription factors. However, TFAM is expected to bind to the mitochondrial genome, which has very different characteristics from the nuclear genome. In addition, it is predicted to bind both in the classical localized manner [36] as well as broadly across the mitochondrial genome. As a result, metrics for evaluating nuclear transcription factors are not well-suited for analysis of TFAM binding data. We therefore examined the fraction of sequencing reads in our libraries mapping to the mitochondria as a proxy for the enrichment of TFAM binding events. Strikingly, between 30% and 75% of TFAM ChIP-seq reads mapped to the mitochondrial genome, while less than 2% of reads mapped to the mitochondrial genome in the input samples, indicating that our TFAM ChIP-seq datasets are indeed highly enriched for TFAM binding events (Figure 1B). We note that 75% ChIP enrichment is extremely high (in fact, practically unprecedented) for any transcription factor dataset [35], thus underscoring the high experimental quality of our datasets.

**Figure 2:**
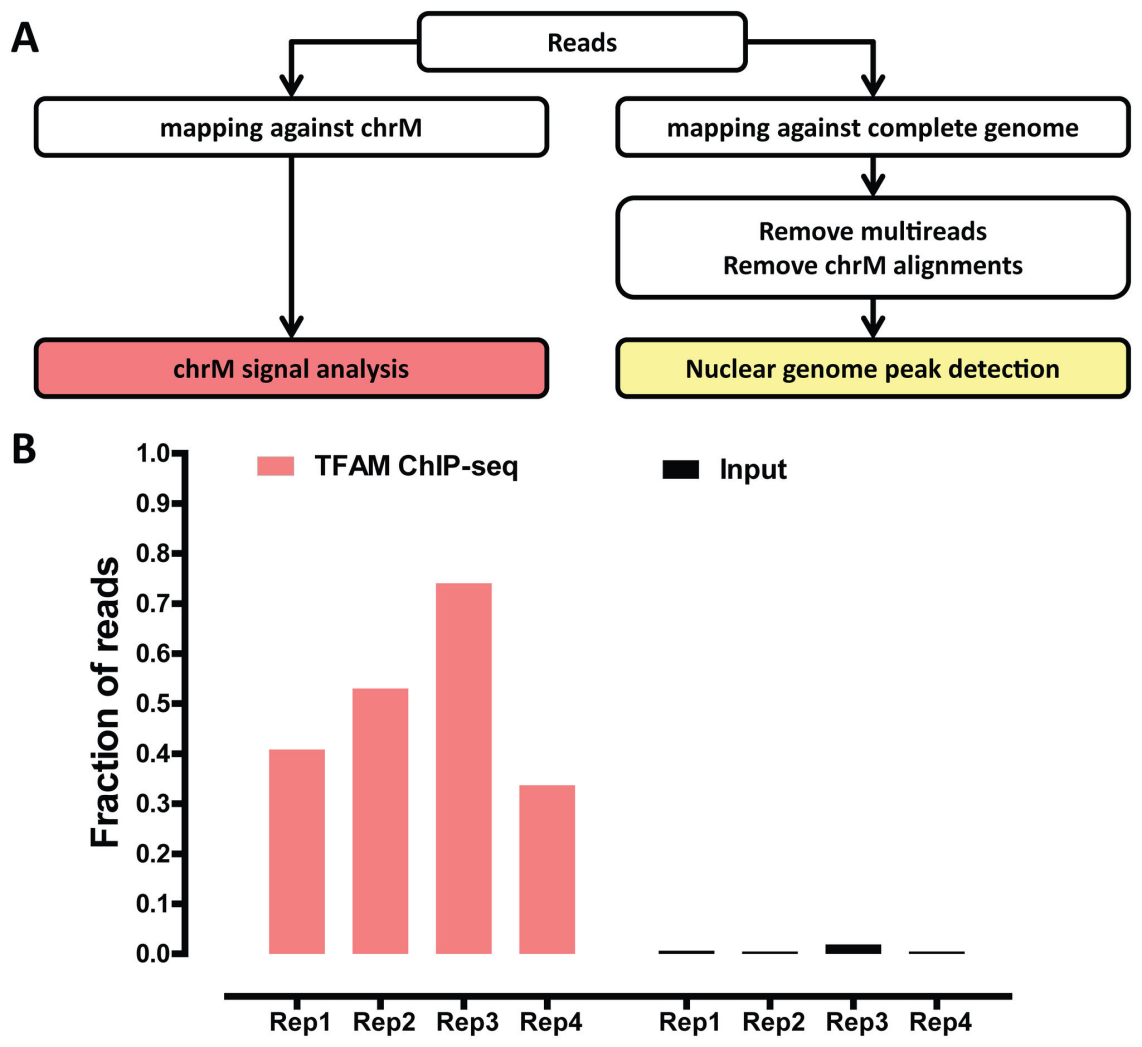
ChIP-seq analysis of genome-wide TFAM binding. (A) Overview of computational processing of data. Reads were trimmed to 36 bp and then either mapped against the mitochondrial genome (ChrM), or the complete hg19 version of the genome. After removing multireads and alignments to the mitochondrial genome, peaks in the nuclear genome were called using MACS2. (B) The proportion of sequencing reads mapping to chrM in ChIP and input datasets. All replicates of the ChIP-seq resulted in at least 30% of reads mapping to the mitochondrial genome, much greater than the 0.4-1.9% of reads mapping to mtDNA in the input datasets. Replicates 1-3 were performed using the 20G2C12 antibody, while Replicate 4 was performed using the 20F8A9 antibody.

Because partial copies of the mitochondrial genome are also present in the nuclear genome, not all reads originating from mtDNA can be mapped uniquely. Therefore, we characterized TFAM binding to mtDNA and to the nuclear genome separately. We analyzed mitochondrial binding events by aligning sequencing reads to the mitochondrial genome alone (restricting our analysis to reads mapping perfectly without any mismatches to further increase mapping accuracy), and analyzed binding to the nuclear genome by aligning only the reads which did not map to the mitochondrial genome, as outlined in Figure 2A. For a standard nuclear transcription factor, this approach may cause some reads originating from the nuclear genome to artificially map to the mitochondrial genome. However, given that TFAM is known to bind to the mitochondrial genome and the extremely high enrichment for TFAM binding to mtDNA in our TFAM ChIP-seq libraries, this should not be a significant confounding factor.

### TFAM coats the mitochondrial genome

As discussed above, TFAM has not only been proposed to bind specifically to well-defined binding sites in the D-loop, but has also been suggested to play a nonspecific packaging role in the nucleoid that is essential for mtDNA integrity. However, little is known about the pattern of non-specific binding of TFAM to the mitochondrial genome. Localized binding at the D-loop and diffuse binding across the rest of the genome are expected to result in distinct ChIP-seq signal profiles. Localized, “point-source” binding to DNA results in an asymmetric distribution of reads mapping to the forward and reverse strand around the binding site of the protein [36,37], while diffuse binding does not produce such strand asymmetry.

**Figure 3:**
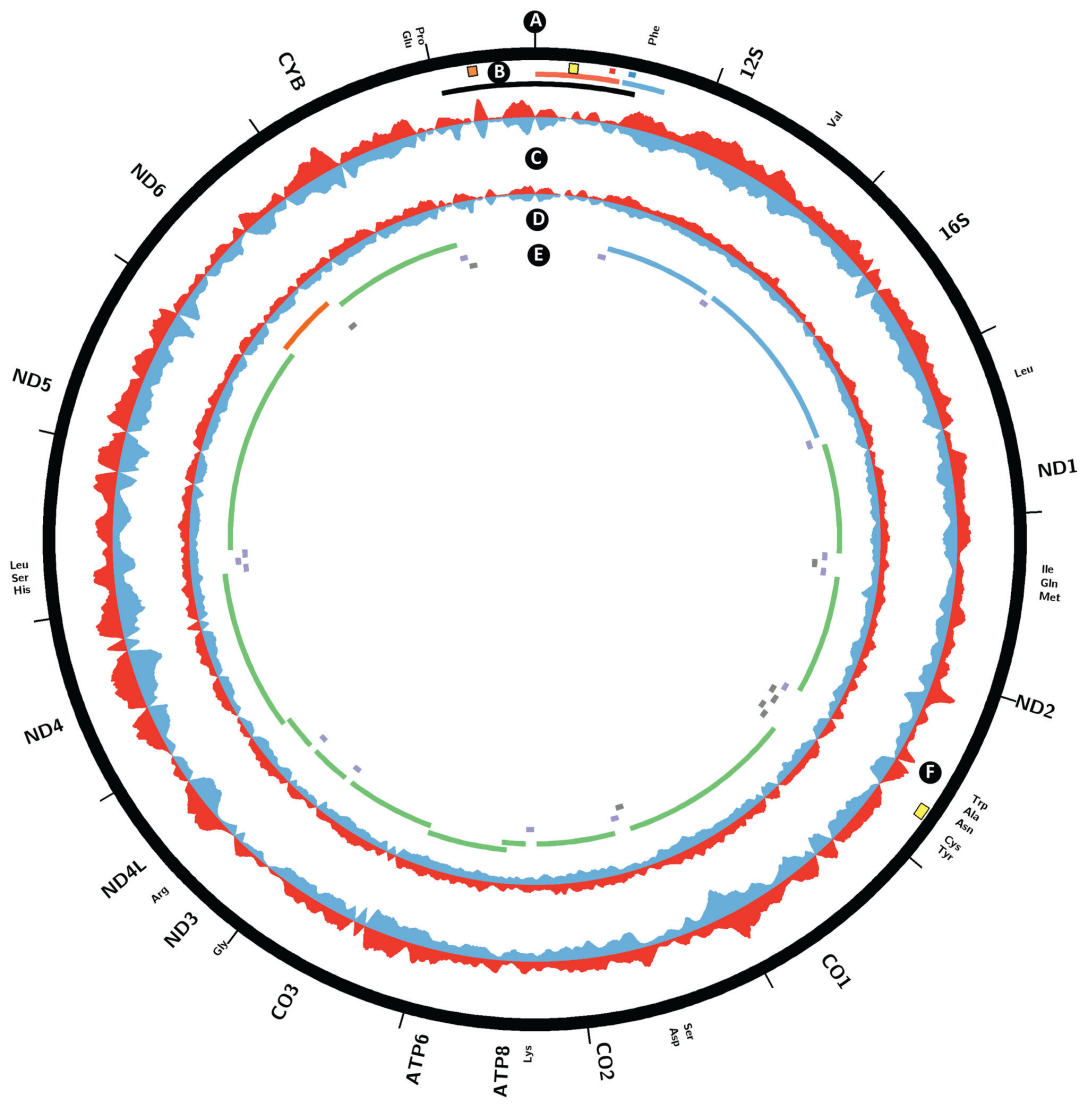
Coating of the mitochondrial genome by TFAM in HeLa cells. Circos plot of plus strand and minus strand TFAM ChIP-seq and input read density signal over chrM. (A, E) Annotation of protein coding (green on forward/heavy strand, red on reverse/light strand), ribosomal RNA (blue) and tRNA (blue on forward/heavy strand, grey on reverse/light strand) transcripts. (B) D-loop (black), LSP promoter (large red tile), known LSP TFAM binding site (small red tile), HSP promoter (large blue tile), known HSP1 TFAM binding site (small blue tile), and origins of heavy strand replication (Ori-b, orange tile; O_H_, yellow tile). (C) TFAM ChIP-seq signal on forward (red) and reverse (blue) strands. (D) Input signal on forward (red) and reverse (blue) strands. (F) Origin of light strand replication (yellow tile). Note that the input signal is exaggerated 60-fold relative to the ChIP-seq signal in order to visualize coverage irregularities. The signal from the TFAM ChIP-seq largely follows that of the input, indicating generalized binding across the mitochondrial genome.

To characterize TFAM binding to mtDNA, we examined the forward and reverse strand read distribution after mapping TFAM ChIP-seq and input library reads to the mitochondrial genome. Strikingly, we did not observe regions of obvious enrichment and strand asymmetry in the D-loop; in particular, we did not see specific binding at the predicted HSP1 and LSP sites. On the whole, the TFAM ChIP-seq signal was broadly distributed over the whole mitochondrial chromosome, and while coverage was not perfectly uniform, the amplitude of the non-uniformity was not significant, and the signal profile closely tracked that of the input sample (Figure 3). The low level of non-uniformity likely results from sequencing biases, which has been documented to skew coverage [38,39]. Because our libraries were carefully size-selected for fragments in the 200 bp range, discrete TFAM binding sites would be expected to yield discrete signal localizations. Therefore, we interpret these results as evidence for the uniform coating of the whole mitochondrial genome by TFAM. We observed one region of apparent localized enrichment exhibiting strand asymmetry in the ND2 ORF near the origin of light strand replication (O_L_) (Figure 3F), which we discuss in the Discussion section.

To further verify our results, we carried out ChIP-seq against TFAM with a second TFAM monoclonal antibody, 20F8A9. We obtained similar results (Figure S1) and found significant correlation between the 20F8A9 dataset and the three datasets obtained from the 20G2C12 antibody datasets (p < 0.0001).

### No evidence for binding to the nuclear genome

**Figure 4:**
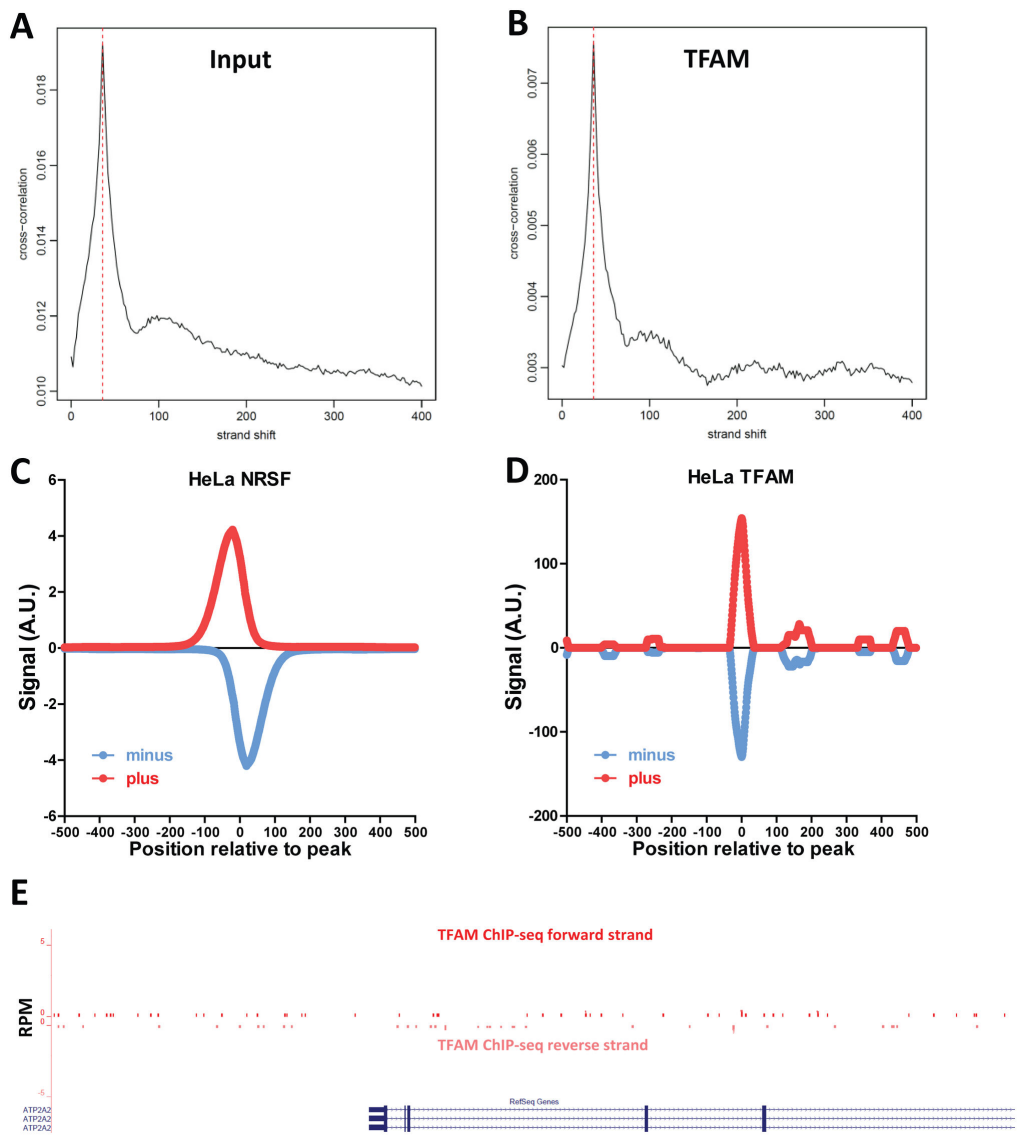
Absence of TFAM binding to the nuclear genome. (A) Cross-correlation plot of input DNA computed over the nuclear genome. (B) Cross-correlation plot of TFAM ChIP-seq computed over the nuclear genome. (C) Distribution of ChIP-seq reads mapping to the plus and minus strand around called binding sites in a ChIP-seq dataset for the NRSF transcription factor [51] in HeLa cells, generated by the ENCODE consortium [52]. (D) Distribution of TFAM ChIP-seq reads mapping to the plus and minus strand around called binding sites indicates lack of real binding sites. (E) No ChIP-seq enrichment around the promoter of the SERCA2/ATP2A2 gene, previously suggested to be a TFAM target.

Previous studies have suggested that TFAM can be found in the nucleus and that it modulates the transcription of nuclear genes. In rat neonatal cardiac myocytes, TFAM was found to bind to the promoter of SERCA2, the homolog of human sarco(endo) plasmic reticulum calcium-ATPase 2 (ATP2A2), and was implicated in regulating its transcription [40]. Given the extremely high degree of TFAM binding enrichment in our datasets, any robust nuclear TFAM binding events should be readily detectable. To analyze nuclear binding, we excluded all sequencing reads mapping to the mitochondrial genome and used the resulting set of reads to identify putative TFAM binding sites. We first looked for significant global read clustering using cross-correlation between reads mapping to the forward and the reverse DNA strands [35,36]. Cross-correlation plots for input samples and for TFAM ChIP-seq datasets were indistinguishable from each other (Figure 4A,B). Next, we called putative TFAM binding sites using MACS2 [41]. Using default settings (corresponding to a q-value cut-off of 10^-2^), we identified 72, 137 and 153 sites respectively for the three replicates generated with antibody 20G2C12, and a single site for the 20F8A9 antibody. However, manual inspection of each of the identified sites revealed that all were likely to represent artifacts, mostly associated with repetitive DNA sequences, as none had the expected strand asymmetry of read distribution around a binding site. Instead, the two strand profiles at each site were identical (summarized in Figure 4D, with the classic nuclear transcription factor NRSF shown for comparison in Figure 4C), and numerous unmappable regions and repetitive elements were present in the immediate vicinity of many of the called sites. Inspection of the ATP2A2 gene revealed no TFAM enrichment neither in the promoter region nor anywhere else in the neighborhood of the gene (Figure 4E). Furthermore, we do not detect nuclear localization of TFAM in our cells (Figure 1C). Therefore, in HeLa cells under normal growth conditions, we find no evidence for specific binding of TFAM to nuclear target genes.

## Discussion

Previous in vitro studies have suggested that TFAM binds specifically to LSP and HSP1, and that it may also bind nonspecifically in a phased manner. Furthermore, evidence has been presented for its nuclear localization and action as a canonical nuclear transcription factor in rat neonatal cardiac myocytes. However, no direct genome-wide measurements of TFAM binding have been previously reported. Our TFAM ChIP-seq data reveal very high enrichment for reads mapping to the mitochondrial genome, but a binding pattern that largely mirrors the read distribution observed in the input DNA, suggesting broad, non-specific binding to mitochondrial genome. This pattern is highly reproducible, indicating that the average population-wide state of TFAM-mtDNA interactions is stable. We found no correlation between irregularities in TFAM signal distribution and characteristics of the mitochondrial genome such as GC content (data not shown). Thus, we conclude that TFAM binds to the mitochondrial genome nonspecifically and without bias when cells are grown under typical culture conditions. Although we do not observe the synchronized phased binding seen in in vitro studies, we cannot rule out a model where individual mtDNAs have such a pattern of binding initiating from a non-universal nucleation site.

Strikingly, we did not observe localized enrichment of binding at the known LSP and HSP1 TFAM binding sites. Peak patterns mirrored that of the input in these regions, and no ChIP-seq peaks displaying the canonical strand asymmetry in read distribution were observed. This finding can be explained by a model in which the interaction of TFAM with the LSP and HSP1 binding sites is relatively transient and infrequent compared to a more stable non-specific association with the genome in its packaging state.

We did detect one site in the genome exhibiting the characteristics of a specific, localized ChIP-seq peak, centered at 5175 bp in the ND2 ORF. The localized nature of the ChIP signal at this site suggests higher occupancy of TFAM. This peak localizes to 546 bp upstream of the O_L_. Strikingly, TFAM has previously been localized 520 bp upstream of the O_L_ of rat mtDNA [42–44]. We found no sequence similarity between the rat and human sites, and in general this region of the mtDNA genome shows low homology between the two species. Further work will be required to understand the significance of this putative TFAM binding site.

Finally, analysis of all datasets for TFAM binding to the nuclear genome yielded no hits distinguishable from common ChIP-seq artifacts. Although Watanabe et al. observed regulation of the SERCA2 gene in rat myocytes, we did not detect TFAM binding at the promoter of its ortholog in humans. Previous studies have shown nuclear localization of TFAM in rat hepatoma cells [45], as well as an alternate isoform of TFAM in mouse testis nuclei [46]. We have thus far been unable to detect nuclear TFAM localization in HeLa cells (Figure 1C), suggesting that nuclear localization and transcriptional regulation may be cell type or perhaps species-dependent. ChIP-seq in different cell lines may be able to detect such nuclear interactions.

We demonstrate here the first high-resolution ChIP-seq analysis of TFAM binding to the mitochondrial genome. Aside from generalized, largely non-specific binding across the mitochondrial genome, we detected a putative specific binding site upstream of the origin of light strand replication. We do not observe the expected binding at the known HSP1 and LSP sites, nor did we identify any nuclear binding sites. An area that remains to be explored is the dynamic nature of TFAM-DNA interactions with respect to both the nuclear and mitochondrial genomes. ChIP-chip on the yeast mitochondrial genome has shown that metabolic changes can lead to differential binding of the yeast TFAM homolog, Abf2p [47]. It is possible that such remodeling also occurs in the mammalian system, and further studies will provide insight into the dynamic nature of the mtDNA-protein interactions within the nucleoid that serve to protect its integrity.

## Materials and Methods

### Cell growth and treatment

HeLaS3 cells were cultured in Dulbecco’s modified Eagle’s medium (DMEM, Invitrogen #11995) containing 10% bovine serum (Invitrogen #16170), penicillin and streptomycin, and additional L-glutamine (2mM). Cells were fed 24 hours before harvest for ChIP-seq, which was performed at 80-90% confluency.

### Antibody Production and characterization

Antibodies were produced by the Caltech Monoclonal Antibody Facility and raised against the full-length TFAM protein in mouse. Immunoprecipitation with 20G2C12 and 20F8A9 TFAM antibodies and Myc antibody (Santa Cruz #sc-40) was performed according to established protocols using M-280 sheep anti-mouse Dynabeads (Invitrogen #11201D). Immunoblotting of IP products was performed using a monoclonal TFAM 18G102B2E11 antibody, also custom generated, at 1:2000, with goat anti-mouse HRP antibody (1:10,000, Jackson ImmunoResearch #115-056-003). Immunoblotting of HeLa whole cell lysate with 20G2C12 was performed at a 1:200 dilution and with goat anti-mouse HRP antibody.

### Immunocytochemistry

HeLa cells cultured as described above were plated onto poly-lysine coated glass coverslips 48 hours prior to fixation in formaldehyde and permeabilization with 0.1% Triton X-100. For colocalization of TFAM to mitochondria, 20G2C12 or 20F8A9 antibodies were used at 1:10 in conjunction with PPIF at 1:200 (ProteinTech #18466-1-AP). Secondary antibodies were goat anti-mouse AF488 (1:500, Invitrogen #A11001) and donkey anti-rabbit AF546 (1:500, Invitrogen #A10040). Cells were also stained with DAPI to visualize nuclei. Immunocytochemistry to visualize colocalization of mitochondrial nucleoids and TFAM was performed sequentially due to both antibodies being raised in mouse. Sequential immunostaining yielded no background fluorescence due to cross-antibody reactivity (data not shown). Order was as follows: anti-TFAM antibody (1:10); goat anti-mouse AF488 (1:500, Invitrogen #A11001); anti-DNA antibody (1:25, Millipore #CBL186); goat anti-mouse AF555 (1:500, Invitrogen #A21426), DAPI. Images were acquired with a Zeiss LSM 710 confocal microscope with PlanApochromat 63X/1.4 oil objective. Z-stack acquisitions were converted to maximum z-projections using ImageJ software.

### Chromatin immunoprecipitation and sequencing

ChIP experiments and preparation of DNA for sequencing were performed following standard procedures [48] with some modifications. Cells were fixed for 10min at RT in 1% formaldehyde, harvested using a cell scraper, washed once in ice-cold PBS, and resuspended in RIPA buffer with protease inhibitor. The sample was then sonicated using a 3.2mm microtip (QSonica Sonicator 4000) at 30s on/30s off intervals and 40% amplitude for 180min while in a -30°C 3:1 isopropanol and water bath containing dry ice. Subsequent steps were performed as per the standard protocol. DNA was size-selected during library building to an average fragment size of 200bp. Libraries were sequenced using Illumina GAIIx and Illumina HiSeq 2000. Sequencing data is available under GEO accession record GSE48176.

### Sequencing data processing and analysis

Sequencing reads were trimmed down to 36 bp and then mapped against either the female set of human chromosomes (excluding the Y chromosome and all random chromosomes and haplotypes) or the mitochondrial genome alone, using the hg19 version of the human genome as a reference. Bow tie 0.12.7 [49] was used for aligning reads, not allowing for any mismatches between the reads and the reference. ChIP-seq peaks were called using MACS2 [41] with default settings except for the mfold parameter, which was lowered to (2,30). Circos plots were generated using Circos version 0.60 [50]. Additional data processing was carried out using custom-written python scripts. ENCODE data was downloaded from the UCSC browser (http://hgdownload-test.cse.ucsc.edu/goldenPath/hg19/encodeDCC/wgEncodeHaibTfbs) and its use here complies with its terms of usage. Pearson correlation coefficient, t-test, and p values were calculated using embedded and custom Microsoft Excel functions.

## Supporting Information

Figure S1 **Comparison of profiles of TFAM binding to mitochondrial genome.**
Circos plots of TFAM ChIP-seq experiments: (1) 20F8A9 antibody ChIP-Seq; (2) 20G2C12 replicate 1; (3) 20G2C12 replicate 2; (4) 20G2C12 replicate 3. Read profiles are very similar across replicates and antibodies.
(TIF)
