# Supplementary figures and images for: Genome-Wide Analysis Reveals Coating of the Mitochondrial Genome by TFAM
Source: PLoS One. 2013 Aug 26;8(8):e74513. doi: 10.1371/journal.pone.0074513 (PMC3753274; doi:10.1371/journal.pone.0074513)

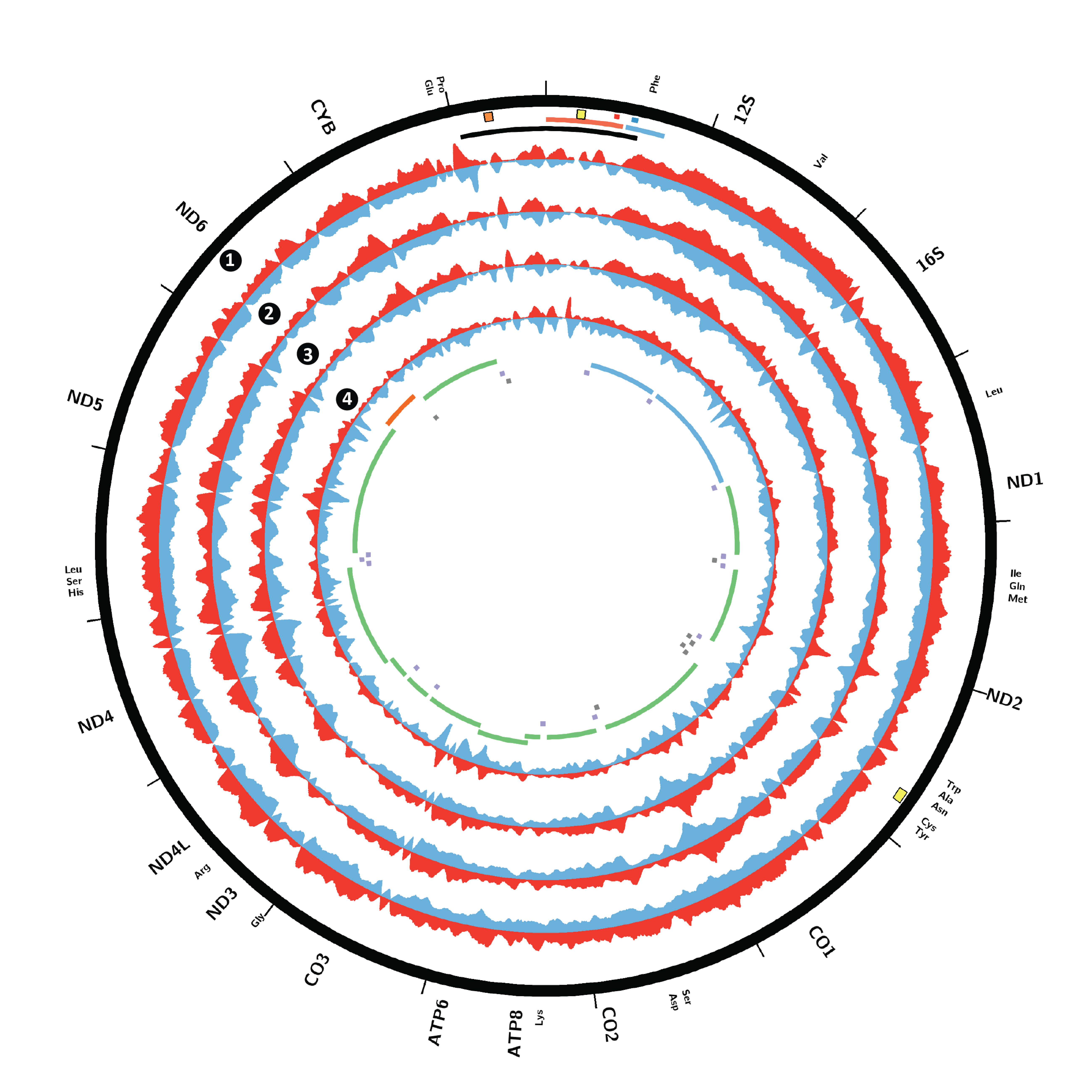

Supplement: Figure S1 — Comparison of profiles of TFAM binding to mitochondrial genome. Circos plots of TFAM ChIP-seq experiments: (1) 20F8A9 antibody ChIP-Seq; (2) 20G2C12 replicate 1; (3) 20G2C12 replicate 2; (4) 20G2C12 replicate 3. Read profiles are very similar across replicates and antibodies. (TIF) [file pone.0074513.s001.tif]
